# A Study of the Flexural Behavior of Fiber-Reinforced Concretes Exposed to Moderate Temperatures

**DOI:** 10.3390/ma14133522

**Published:** 2021-06-24

**Authors:** Marta Caballero-Jorna, Marta Roig-Flores, Pedro Serna

**Affiliations:** 1 ICITECH—Institute of Concrete Science and Technology, Universitat Politècnica de València, 4N Building, Camino de Vera s/n, 46022 València, Spain; pserna@upv.es; 2 Department of Mechanic Engineering and Construction, Universitat Jaume I, Av. Sos Baynat s/n, 12071 Castelló de la Plana, Spain; roigma@uji.es

**Keywords:** aging, durability, fiber-reinforced concrete, macrosynthetic fibers, residual flexural strength, temperature

## Abstract

The use of synthetic fibers in fiber-reinforced concretes (FRCs) is often avoided due to the mistrust of lower performance at changing temperatures. This work examines the effect of moderate temperatures on the flexural strengths of FRCs. Two types of polypropylene fibers were tested, and one steel fiber was employed as a reference. Three-point bending tests were carried out following an adapted methodology based on the standard EN 14651. This adapted procedure included an insulation system that allowed the assessment of FRC flexural behavior after being exposed for two months at temperatures of 5, 20, 35 and 50 °C. In addition, the interaction of temperature with a pre-cracked state was also analyzed. To do this, several specimens were pre-cracked to 0.5 mm after 28 days and conditioned in their respective temperature until testing. The findings suggest that this range of moderate temperatures did not degrade the behavior of FRCs to a great extent since the analysis of variances showed that temperature is not always a significant factor; however, it did have an influence on the pre-cracked specimens at 35 and 50 °C.

## 1. Introduction

Fiber-reinforced concrete (FRC) is a composite material containing disperse fibers inside a cementitious matrix. The fibers give the mixes improved mechanical capacity, greater ductility and durability [1,2]. FRC is already used worldwide, and fibers have been gradually included in standards and codes [3,4,5]. However, these codes are generally based on experiences with steel fibers (the most extended fiber type used in structural applications) and therefore do not cover all types of fiber material on the market. In particular, the FIB MC2010 [5] expose warnings for those with a Young’s modulus that is affected by time or thermo-hygrometric phenomena, as synthetic fibers. Synthetic fibers have emerged in different shapes, sizes and compositions and in recent years have gained popularity, especially polypropylene fibers.

Synthetic fibers are considered to be a potential concrete reinforcement having enhanced corrosion and chemical resistance [6,7,8]. However, their role in structural uses is often considered limited, especially when compared to steel fibers. Criticism of their durability and contribution in structural uses are based on a low elasticity modulus (210 GPa of steel vs typically <10 GPa for polymeric fibers), a density slightly lower than that of water, and a melting point around 160 °C [9]. However, macrosynthetic (MS) fibers can be used in some specific applications where those disadvantages do not apply because cracking is not expected, e.g., in applications where a serviceability limit state (SLS) is not important, as a minimum shear reinforcement or in structures where FRC creep does not influence ultimate limit state (ULS) capacity [10,11]. Some authors [12] claim that the use of synthetic fibers as reinforcement should not be disregarded as long as additional factors are considered during the design, including initial crack opening and environmental conditions. Thus, the adoption of MSFRC could be promoted for these purposes as long as further investigations into their short- and long-term behavior are carried out to verify their performance and mitigate mistrust regarding the use of synthetic fibers.

As previously mentioned, one of the main drawbacks of synthetic fibers is that their properties are influenced by hygrothermal phenomena [5,13]. Temperature changes can affect their chemical, physical and mechanical properties, which can lead to dramatic degradations and loss of bearing capacities and in turn to the degradation of the integrity of the concrete. To date, this topic is a point of on-going discussion at the technical and scientific level. While it seems clear that MSFRCs exposed to temperatures close to fiber material’s melting point will suffer strong property degradation, only a few studies have analyzed the effect of moderate temperatures on MSFRCs. The range of moderate temperatures includes those below 80 °C, which can be found in severe operating conditions in some industrial applications, such as those related to energy [14]. Extensive literature has been published on FRC and MSFRC elements at extremely high temperatures in simulations of accidental fires [15,16,17,18], but those temperatures are beyond the scope of this work.

In this range of moderate temperatures, the concrete matrix already experiences property changes. For instance, Joos and Reinhardt [14] reported that different concrete types have shown permeability increases of 13–62% when the temperature increased from 20 to 50 °C, and of 3–55% from 50 to 80 °C. The diffusivity also increased by 10–21% from 20 to 50 °C and by 8–21% from 50 to 80 °C. The change in these two properties was very different depending on the type of concrete. Other researchers also found degradation in the reduction of the modulus of elasticity when concrete was heated from 60 to 80 °C [19], while other works indicated that increasing the temperature from 20 to 80 °C can increase the compression strength of the concrete matrix [20]. Another work studying the microstructure of cement pastes at 20, 40 and 60 °C [21] demonstrated that these temperatures had already produced changes in pore structure and water transport.

Several researchers have also investigated MSFRCs exposed to moderate temperatures. On the one hand, Buratti and Mazzotti [22] investigated the effect of moderate variations (20–50 °C) on short- and long-term behavior in cracked MSFRC structural elements. They found that moderate temperatures reduced the short-term residual strength of some MSRFCs by up to 20%. The study concluded that temperature should always be considered as an important factor for the long-term behavior of MSFRCs. Later on, these authors also compared the influence of temperatures from 20 to 40 °C on the behavior of steel fiber-reinforced concrete (SFRCs) and MSFRCs [23]. They concluded that, for a large-crack mouth opening displacement (CMOD) larger than 1.5 mm, the strength variation of the SFRC was about 3% while that of the MSFRCs was about 10%. For smaller CMODs, temperature effects were negligible.

Richardson and Ovington [24] examined how temperature variation (20 ± 40 °C) affected the properties of concrete with steel and synthetic fiber additions. The results showed that all beams experienced a flexural strength reduction when exposed to 60 °C compared to ambient temperature. For all beams tested at −20 °C, flexural strength increased, which was thought to be produced by freezing. The SFRC performed the best within the parameters of this experimental campaign although synthetic fibers were also effective at providing post-crack flexural toughness at the tested dosages.

On the other hand, other experimental investigations demonstrated that MSFRC mechanical properties are affected at temperatures mainly above 100 °C. For instance, Strauss Rambo et al. [25] showed that the effect of temperature on the mechanical behavior of MSFRCs in the Barcelona Test was very similar to that known for conventional concrete concerning the loss of mechanical strength and elastic modulus up to 100 °C. These last works are in agreement with the information from the macrosynthetic fiber supplier, who guarantees performance below the melting point. Moreover, Hannat [26] concluded that synthetic fibers are durable inside the concrete matrices, and these have been shown to give a post-crack behavior over many years.

Additionally, the different test setups used in the aforementioned campaigns and the wide contrast in their parameters (e.g., dimensions of the tested elements, selected temperatures) explain the variability of the results, which generates high interest in standardizing testing methodologies to analyze the effect of moderate temperature variations on the behavior of MSFRCs This standardization will potentially provide new insights on this topic, answering the disagreements about the performance of this material between different suppliers, field engineers and researchers [27,28].

In summary, the influence of moderate temperatures on the mechanical performance of MSFRCs is not fully understood, and several points require further investigation. The aim of this study is to determinate whether MSFRCs may be safely used in several environments and conditions (in uncracked and pre-cracked states) between 5 and 50 °C. This research simulates realistic environments where MSFRCs with polypropylene fibers may undergo a loss of long-term serviceability after two months’ exposure to moderate temperatures. Since this study focuses on service conditions, the temperatures selected were below the melting point of the material, and the test methodology was adapted to maintain the temperature during the test despite the difficulty of manipulating the conditioned specimens [29]. This focus will add information regarding MSFRC behavior under service conditions, which will complement most of studies in the literature that were performed after a degradation process and tested at room temperature.

## 2. Materials and Methods

### 2.1. Materials

The concrete selected in this study corresponds to a mix commonly used in the precast industry, having a compressive strength of 35 MPa and a 10 mm maximum aggregate size. The dosage was designed with OPC CEM II/A-M (V-L) 42.5 R, two coarse aggregates (4–6 mm and 6–10 mm), natural sand (0–4 mm) and limestone filler. A polycarboxylate-based superplasticizer (SikaViscoCrete-5980) was employed to guarantee the workability of the mixtures. The effective water/binder (w/b) ratio was 0.55. Table 1 shows the concrete composition for the FRCs in this study. The fiber contents were selected to obtain comparable residual strength values. The fibers contents selected were 1 and 0.4% by volume for the macrosynthetic and steel fibers, respectively.

**Figure 1:**
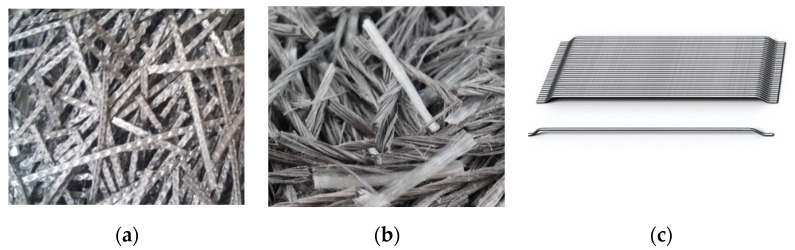
Photos of the fibers used in the study: (**a**) type P1; (**b**) type P2; and (**c**) type S.

Two types of macrosynthetic fibers were introduced into the base concrete: one, a 35 mm corrugated length (type P1, Figure 1a); the other, a 54 mm intertwined length (type P2, Figure 1b). Both are macrostructural synthetic fibers made of polypropylene with different compositions. The P1 fibers were a copolymer of polypropylene (polypropylene, polyethylene and various additives). The P2 fibers were derived from polypropylene (polyolefin polymer). Hooked-end steel fibers of 48 mm were used as the reference fiber (type S, Figure 1c).

These fibers are commonly used and their technical properties, given by their suppliers, are collected in Table 2.

### 2.2. Methods

**Figure 2:**
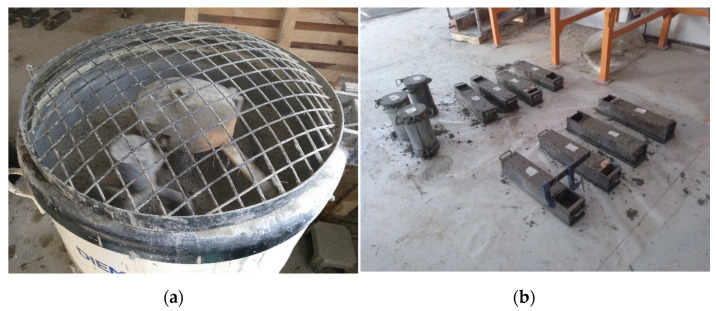
(**a**) Production of concrete and (**b**) specimens produced in a batch of this program.

The experiment was carried out to evaluate the effect of moderate temperatures (5, 20, 35 and 50 °C) on the mechanical behavior of MSFRCs, in particular their residual flexural strengths and toughness. All concrete mixes were manufactured in a DIEM WERKE model DZ 180V mixer and produced by the same sequence to ensure reproducibility of results (Figure 2a). First, coarse aggregates and sand were added and pre-mixed for two minutes. Then, cement and filler were added and mixed for two additional minutes. Afterwards, water was introduced, and then all the materials were mixed for one minute. Finally, following the gradual addition of the fibers, a superplasticizer was added, and a final mixing was applied for 5 min. Afterwards, the concrete was poured into the molds according to UNE EN 12350-1 (Figure 2b).

**Figure 3:**
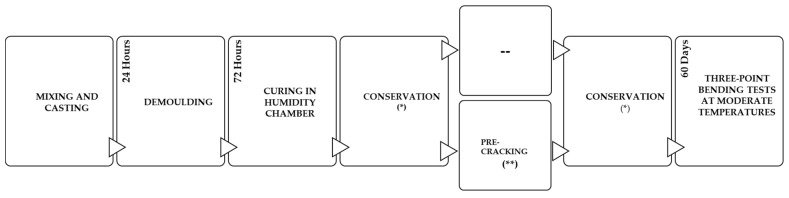
Summary of the procedure. (*) Conditions described in Table 3; (**) Depending on the specimens.

One day after their production, the specimens were demolded and stored for three days in a humidity chamber at 20 °C and 95% relative humidity. They were then moved to their respective conservation condition until testing at 60 days. At 28 days of conservation, some of the specimens were pre-cracked (maintaining the target temperature during the test) and stored again in their respective environment. The procedure is represented in Figure 3.

Table 3 displays the conservation conditions, for which the ambient temperature of the Spain’s Mediterranean climate (20 °C) was used as a reference. Temperatures of 5 and 35 °C can be found in that climate, while 50 °C could represent an industrial application involving warm waters. Periodical measurements of the temperature were conducted to verify the conditions.

**Figure 4:**
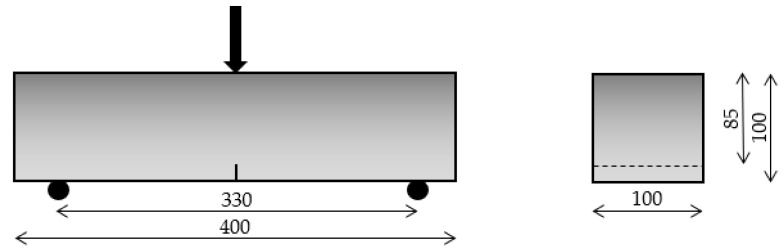
Scheme of the adapted three-point bending test.

Given that there is no standard protocol for studying the influence of moderate temperatures on FRCs, mechanical behavior was evaluated by means of a three-point bending test on notched beams. These tests were performed in an INSTRON 3382 machine, following a methodology based on UNE-EN 14651:2007+A1:2008 [30] with some modifications. The set-up configuration was scaled down to 2/3 of the standard test. Thus, parameters such as the distance between the support (330 mm), the specimens’ dimensions (100 × 100 × 400 mm³) and the depth of the notch (15 mm) were obtained considering this factor. The scheme of the adapted protocol is represented in Figure 4.

**Figure 5:**
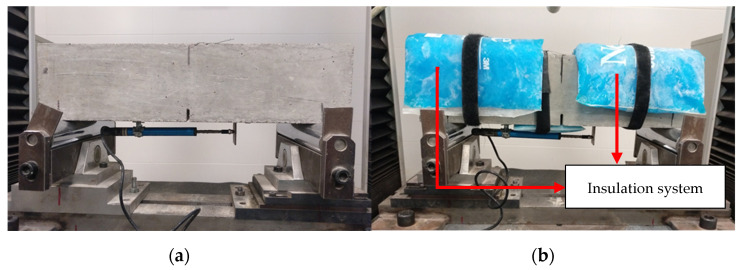
Experimental set-ups used for testing the beams: (**a**) at standard temperature and (**b**) at target temperature with the designed system.

In addition, this modified procedure also included an insulation system during the test to evaluate the influence of the temperatures (5–50 °C) (Figure 5). Reusable cold and hot gel packs were used to maintain the required temperature, while thermal bags were used to insulate the specimens during the tests.

The test was controlled by a CMOD using a linear displacement sensor placed on the bottom face of the specimens. The test was run at a load velocity of 2 mm/min. Then, the data were collected and expressed in stress-CMOD diagrams. The results of flexural strength at the limit of proportionality ($f_{L}$) and at CMOD_j_ ($f_{R,j}$) were calculated using Equations (1) and (2) respectively.

(1) $$f_{L}=\frac{3}{2}\times \frac{F_{j}l}{{\mathrm{bh}}_{\mathrm{sp}}²}\;$$

(2) $$f_{R,j}=\frac{3}{2}\times \frac{F_{j}l}{{\mathrm{bh}}_{\mathrm{sp}}²}$$

where $F_{j}$ is the axial load recorded during the test; l, the distance between supports (330 mm); b, the width of the sample cross-section (100 mm); and h_sp_, the distance between the top of the notch and top of the cross-section (85 mm). The points of CMOD_j_ (j = a, b), where the flexural strength was determined, are as follows: CMOD_a_ = 0.5 mm, and CMOD_b_ = 2.5 mm. Those that were similarly defined in the standard test were considered as reference values [30].

The target crack width was fixed at CMOD_a_ = 0.5 mm for pre-cracked specimens, to simplify the test procedure considering that a 0.5 mm wide crack can simulate a cracked state in an SLS. This was determined in order to investigate if fibers directly exposed to the target temperatures through a cracked section (which may occur in some service conditions) were affected differently to those still protected by the uncracked concrete matrix.

### 2.3. Experimental Program

Table 4 summarizes the experimental program. The code assigned to each group included characters that defined the fiber type, pre-cracking condition (N for uncracked and P for pre-cracked) and temperature. Four prisms per combination were tested. In total, 56 specimens were subjected to three-point bending tests.

**Figure 6:**
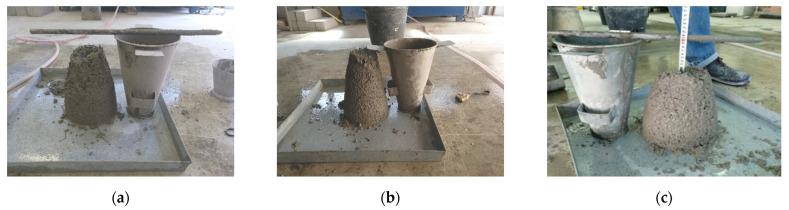
Examples of the visual aspect of the concrete mixes: (**a**) Batch 3, P1 MSFRC; (**b**) Batch 6, MSFRC; and (**c**) Batch 7, SFRC.

The concrete mixes were characterized through compressive strength and workability tests that followed EN 12390-3:2009 and EN-12350-2:2009 standards, respectively. For the compressive strength, three cylinders having a diameter of 150 mm and height of 300 mm were tested per batch, for a total of 24 cylinders. Regarding workability, a single test per mix was performed for a total of 8. Figure 6a–c show example pictures of the slump test. They showed that concrete reinforced with macrosynthetic fibers presented a drier consistency than did those reinforced with steel fibers, despite having the same w/b ratio. This could be explained by the tendency of macrosynthetic fibers, especially P2 fibers, to interweave until they became completely bundled. This, together with the large surface area, caused the cement paste to wrap around them, making these mixes less workable and harder to cast than the rest.

## 3. Results

### 3.1. Characterization Tests

The results of the characterization tests are presented in this section. The objective of the slump test was to verify the workability of the mixes. Table 5 presents the results of the consistency class obtained for each batch. The highest slump values were obtained for mixes reinforced with steel fibers (B 7 and B 8), which exhibited high workability without segregation. On the other hand, a decrease in the slump was seen in those mixes reinforced with macrosynthetic fibers, especially for P2 fibers (B 5 and B 6). This effect could be explained by the high fiber volume and the aspect ratio of this type of fiber, which affects the number per cubic meter in concrete [10]. An MSFRC needs less fiber weight but more fiber volume compared with the SFRC, which affects the consistency of the mixes [31,32]. However, this loss of workability may be overcome by adjusting the amount of plasticizer and using high-energy compaction.

The compressive strength of hardened concrete was tested to determine the uniformity and quality of the different mixes, and because of that it was evaluated only at room temperature. Table 5 summarizes the mean results of the compressive strength tests with their coefficients of variation (CV), calculated from three specimens per batch. The results show similar values for all cases, around 35 MPa. All batches had high homogeneity within each series (CV values less than or around 5%). Variations in compressive strength among batches were assumed to be acceptable and could be explained by intrinsic production variations among specimens. It is important to note that the adding of fibers to the volume contents in this study did not affect compressive strength, which was consistent with other studies [33].

### 3.2. Flexural Strengths at Moderate Temperatures

Three-point bending tests were performed at different temperatures to evaluate the influence of moderate temperatures (5–50 °C) on the mechanical behavior of the FRCs. The procedure made it possible to consider temperature as a factor while it was maintained during a test. Thus, the results were obtained at target temperature, unlike most of the research in the literature which was carried out at room temperature after heating/cooling.

The mean results of the residual flexural strength corresponding to the limit of proportionality ($f_{L}$), CMOD_a_ ($f_{R,a}$) and CMOD_b_ $(f_{R,b}$), and their corresponding CVs are displayed in Table 6 and Table 7 according to the cracked state. It is important to point out that results of $f_{L}$ and $f_{R,a}$ for the pre-cracked condition were obtained at 28 days, whereas the results of $f_{R,b}$ were evaluated at 60 days along with the rest of results. It should be noted that the variability of the results was significant; however, these CV (%) values are frequent when testing residual strength in FRCs [34].

**Figure 7:**
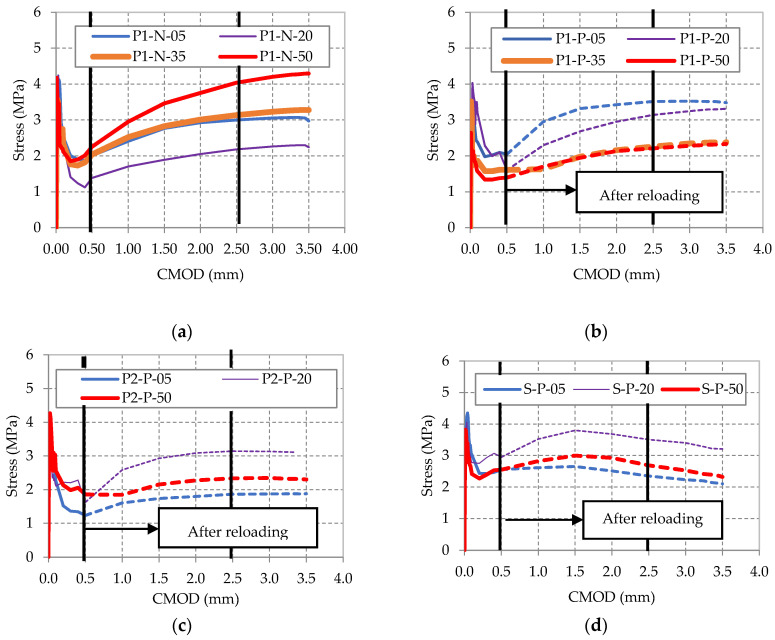
Stress-CMOD curves at different temperatures for each FRC reinforced with: (**a**) P1 (uncracked state), (**b**) P1 (pre-cracked state), (**c**) P2 (pre-cracked state) and (**d**) S (pre-cracked state).

The mean stress-CMOD curves for each group are displayed in Figure 7a–d according to fiber type, temperature and pre-cracked/uncracked condition. Note that each curve is the average of the 4 tested specimens. A representative color was defined for each temperature: blue represents 5; purple, 20; orange, 35; and red, 50 °C. The dashed line represents the data obtained after reloading. The results showed that the selected synthetic- and steel-fiber dosages had comparable residual strength.

Figure 7a,b shows that the pre-cracked condition seemed to affect the residual flexural strength of MSFRCs. Specimens tested in uncracked conditions (Figure 7a) showed higher residual strength than their equivalents tested in a pre-cracked state (Figure 7b). Figure 7a,b also shows that a MSFRC with P1 fibers in pre-cracked conditions obtained a slightly higher flexural residual strength in colder temperatures, with values similar to those obtained for uncracked specimens for warmer temperatures. Figure 7c,d illustrates that an MSFRC with P2 and the SFRC, both in pre-cracked conditions, had the highest residual specimen strength at 20 °C and the lowest at 5 °C.

**Figure 8:**
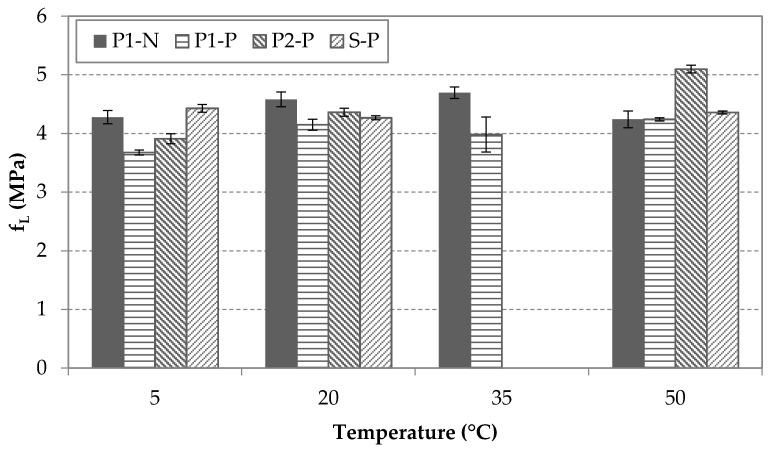
Results of $f_{L}$ for each type of fiber and condition at different temperatures.

**Figure 9:**
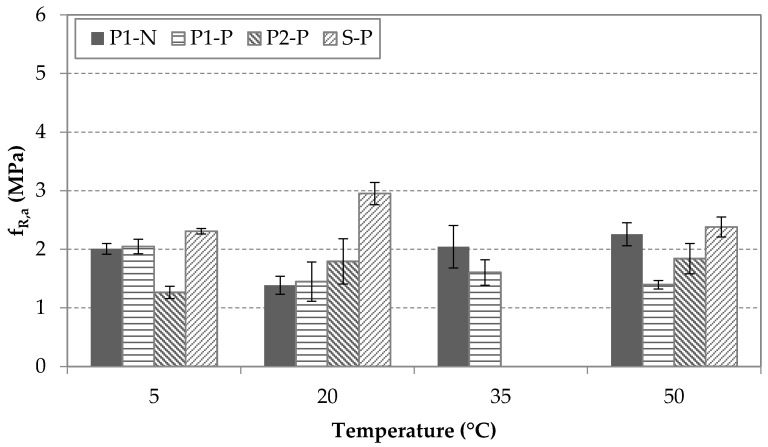
Results of $f_{R,a}\;$ for each type of fiber and condition at different temperatures.

**Figure 10:**
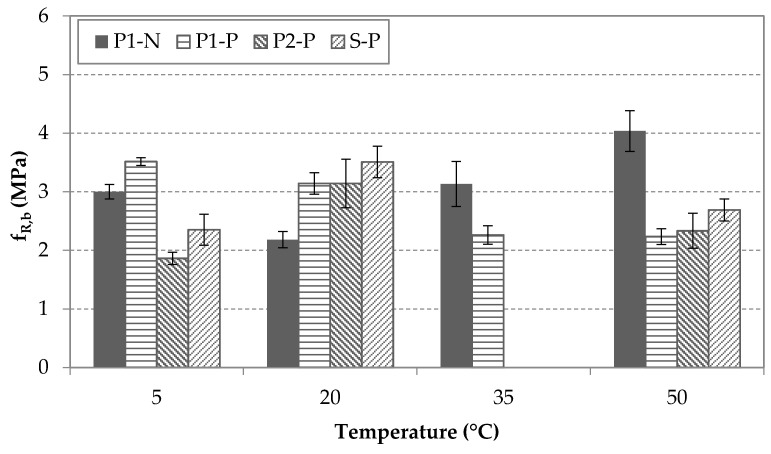
Results of $f_{R,b}$ for each type of fiber and condition at different temperatures.

Figure 8, Figure 9 and Figure 10 display the values of $f_{L}$ and $f_{R_{j}}$ (j = a, b) for each type of fiber, each condition of pre-cracking and each temperature, regardless the age of testing. Figure 8 shows similar results for residual peak-load flexural strengths for S-P and P1-N fibers at temperatures that had no significant effect on the $f_{L}$. In contrast, the P2-P and P1-P specimens displayed a slight rising trend with increasing temperature, which may have been related to an increase in the matrix strength since the $f_{L}$ parameter was heavily related to the matrix’s mechanical properties, mainly compression strength [8]. It may be concluded that the interaction between the pre-cracked condition and temperature may be significant in this case and be worth an in-depth study.

Figure 9 shows the residual flexural strength at CMOD_a_ for the MSFRCs at different target temperatures. P1 and P2 specimens obtained lower $f_{R,a}\;$ strengths than the S specimens did, which was likely caused by the higher elastic modulus of steel fibers. It is noteworthy that the variability measure $f_{R,a}\;$ was higher than in the residual strength results obtained at the limit of proportionality for all groups. Figure 9 shows that the S specimens experienced a slight decrease in $f_{R,a}\;$ for cold and hot temperature compared with the reference temperature (20 °C), but this change was negligible. For P2 specimens, the decrease in temperature may have caused a slight reduction in $f_{R,a}$. However, the values of residual flexural strengths obtained at standard and hot temperatures (20 and 50 °C) did not present notable differences. In the case of the $f_{R,a}\;$ for P1-N, specimens obtained similar residual flexural strength independently of temperature. A descending trend was observed for the pre-cracked group of this type of fiber (P1-P) when the temperature was increasing.

Figure 10 shows the temperature effect on the residual flexural strength $f_{R,b}$ at CMOD_b_ for all groups. This parameter is the one that suffered the highest variation levels in trends and CV (%). The S and P2 specimens exhibited similar behavior for $f_{R,b}$ between 5 and 50 °C. For the specimens reinforced with the macrosynthetic fiber P1, temperature influenced $f_{R,b}$ and additional differences were detected between pre-cracked and uncracked specimens. The residual flexural strengths of the P1-N specimens increased when the temperature rose, but the contrary effect was observed for P1-P specimens. This difference could be explained by the fibers’ being embedded within the uncracked matrix, which could have provided a protective cover that helped minimize susceptibility to environmental effects. Another explanation may be that hydration improved the fiber-matrix bond.

### 3.3. Toughness

Toughness was evaluated to quantify post-crack behavior since this parameter is also considered in FIB MC2010 [5] to determine if an FRC is structural and if differences produced by the conditioning temperature can be detected. To calculate the toughness indices, the area under the stress-CMOD curves (Figure 7) was calculated for four deflection points: (a) up to the first-crack deflection, (b) up to 3.0 times the first-crack deflection, (c) up to 5.5 times the first-crack deflection, and (d) up to 10.5 times the first-crack deflection. The toughness indices were calculated by dividing the aforementioned areas (b), (c) and (d) by the area up to first-crack deflection (a). These indices are named I_5_, I_10_ and I_20_. The average values for each group and the CV are displayed in Table 8.

**Figure 11:**
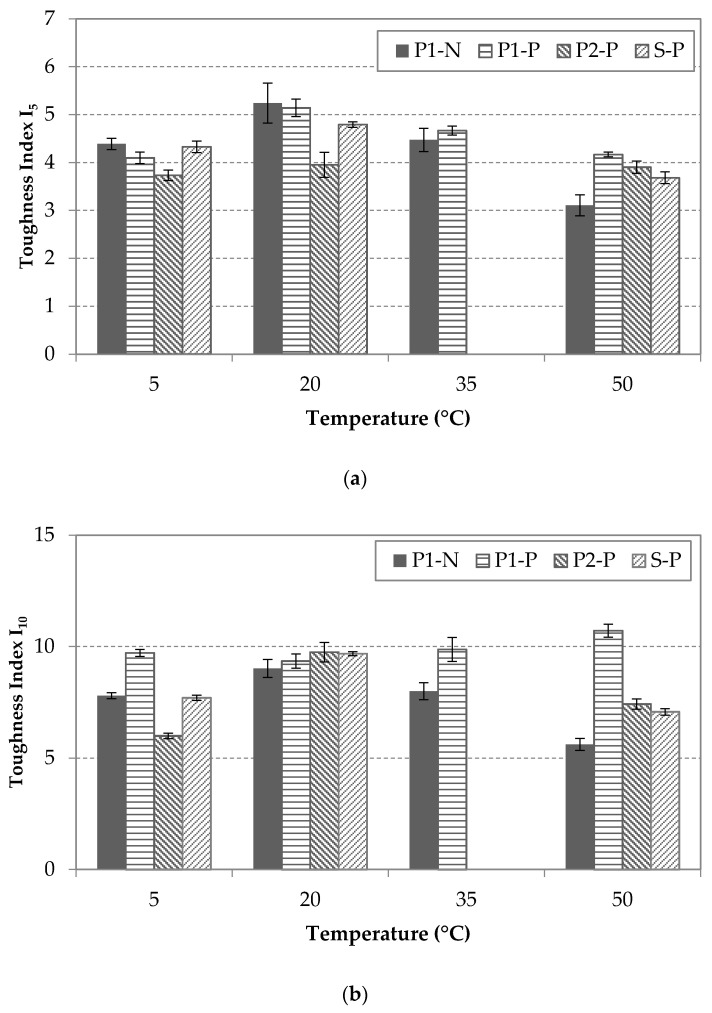

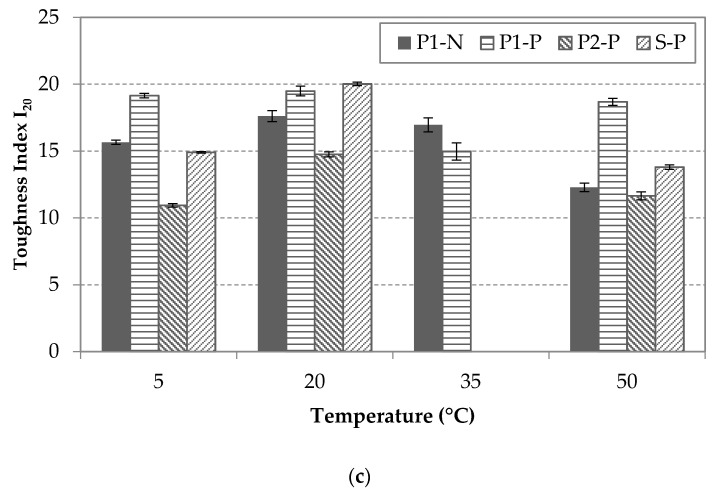
Results of toughness: (**a**) Index I_5_, (**b**) Index I_10,_ and (**c**) Index I_20_ for each type of fiber and condition at different temperatures.

For the toughness results, Figure 11 compares the different indices at different temperatures for each condition and fiber type. As it can be seen for I_5_ in Figure 11a, the temperature influence was not significant, but a slight change could be detected for S-P and P1-N at 50 °C. For I_10_ and I_20_, the toughness trends were similar (Figure 11b,c), reaching their maximum at 20 °C in all the groups, except for P1-P.

## 4. Discussion

### 4.1. Statistical Analysis of the Flexural Strengths at Moderate Temperatures

Variations in flexural strength were within the range of normal variation for CV in the SFRC and MSFRCs [34]. To identify significant differences and relationships among the groups, the results were compared using an analysis of variances (ANOVA) based on the assumption that the data came from a normal distribution.

ANOVA tests were performed to study the influence of moderate temperatures on the average residual flexural strength at different CMODs. The null-hypothesis is that the residual flexural strengths at the different temperatures were equal. Thus, the null hypothesis was rejected when the *p*-value was smaller than 0.05 (95% confidence level). For this purpose, the software Stat graphics was employed to obtain these values. Two independent variables (type of fiber and temperature) were used to perform the analysis, and only the results from the pre-cracked state were used.

All the results from this analysis are collected in Table 9, Table 10, Table 11, Table 12, Table 13, Table 14, Table 15, Table 16 and Table 17. Table 9, Table 10 and Table 11 show the tabulated results from applying the ANOVA analysis to the three types of fibers on $f_{L}$. An effect of temperature factor on $f_{L}$ was observed for the MSFRCs. This influence has limited statistical significance for MSFRC with P1 fibers since the *p*-values are almost at the significance threshold. However, for the SFRC, the effect was not significant.

Table 12, Table 13 and Table 14, summarize the results of applying this ANOVA analysis for the three types of fibers on $f_{R,a}$ For the two FRCs, temperature was not an important factor for $f_{R,a}$. Nevertheless, there was a significant difference in the mean value of $f_{R,b}$ for concretes reinforced with S fibers and P1 fibers (Table 15 and Table 17). For P2 elements, the temperature caused changes in the values of $f_{R,b}$ but were not statistically relevant (Table 16).

### 4.2. Comparison of the Results with Other Studies

The results of this study were consistent with previously published results in the literature but with some differences. Similar to this study, Buratti and Mazzotti [23] found that the SFRC and MSFRCs had different flexural-strength behavior with temperature, but they affirmed that the effect of temperature was less relevant for the SFRC than for the MSFRCs. This affirmation was not confirmed by the data obtained from this work since the temperature factor seemed to affect the SFRC and MSFRCs in a similar way.

Furthermore, they also concluded that the temperature reduced the short-term residual flexural strength of some of the specimens and that temperature should be considered as a factor when designing MSFRCs. However, this study indicated that variations in temperature may cause an increase or reduction in residual flexural strengths after two months, but this variation was not significant for all the cases.

The study of Rambo et al. [25] reported that the temperatures applied under the residual flexural tests in their study (below the melting point) were not capable of altering the mechanical properties of the FRCs. In this study, the conclusions were similar for residual strengths at smaller CMODs, supporting the view that moderate temperatures do not affect the performance of concretes reinforced with polypropylene fibers; however, a limited loss of serviceability of FRCs could be expected for situations with large CMODs.

Regarding the pre-cracked condition (0.2 to 3.5 mm), some authors [35] studied its effect on the long-term performance of fiber-reinforced beams and reported that crack-width directly affected MSFRC behavior. On the other hand, other studies [12] reported that the use of plastic fibers as reinforcement should not be rejected as long as the additional creep or temperature factor is considered in the design. Regarding the influence of the pre-cracking conditions, this study showed that environmental conditions (temperature and humidity) may be considered to a limited extent.

For the toughness indices, values were in the common range for fiber-reinforced concretes: 1 to 6 for I_5_, 1 to 12 for I_10_ and 1 to 25 for I_20_ [36]. Steel- and macrosynthetic fiber-reinforced concretes performed similarly. Of the two polypropylene fibers at the tested temperatures, P1 obtained higher flexural-response values than the P2 fibers did. Regarding the steel-fiber specimens, the results were different that those of other authors who investigated the behavior of SFRCs under flexural loading [37]. They pointed out an increase in toughness when the temperature fell. However, in this study, the SFRC specimen presented a decline in residual strength at low temperatures. In conclusion, the effect of heating and cooling on variations in toughness cannot be considered negligible in the I_20_ index, but this effect should be investigated further.

In short, the outcomes of our research showed that moderate temperatures cannot be considered a crucial factor on the behavior of MSFRCs. Therefore, the option of using structural synthetic fibers should be considered depending on the requirements of their application. These results could extend their use since their remaining residual strength may be enough to ensure safety in common conditions. In any case, their structural capacity could be slightly reduced under long-term extreme temperatures. To reach broader and stronger conclusions, developing this study based on a more representative number of specimens, and of a standard size, is recommended.

## 5. Conclusions

In this work, an experimental investigation was carried out to evaluate the effect of temperature on the mechanical properties of fiber-reinforced concrete containing steel and macrosynthetic fibers (SFRC and MSFRCs). These had been tested at constant moderate temperatures (5, 20, 35 and 50 °C), in uncracked and pre-cracked conditions. The following conclusions can be drawn:

- The outcomes derived from the beams tested in the three-point bending test showed that the post-peak flexural behaviors of the two MSFRCs (with P1 and P2 fibers) and the SFRC were not affected for a small CMOD (CMOD_a_ = 0.5 mm), after being stored for 60 days between 5 and 50 °C. The temperature effect was more noticeable for a larger CMOD (CMOD_b_ = 2.5 mm) for both FRCs and this factor may be considered to have a limited influence.
- The interaction of the pre-cracked condition with its exposure to moderate temperatures was influential at 35 and 50 °C for the MSFRCs with fiber type P1; that is, when the specimens were pre-cracked and exposed to moderately hot temperature. A drop in the residual flexural strengths was detected, but a specific study is recommended to better determine this influence.
- Toughness behavior was studied at these temperatures, and both steel and synthetic fibers provided good mechanical responses at the low and high temperatures in pre-cracked and uncracked states. The influence of the temperatures was similar for all properties regardless of the fiber material, given that that the dosage of each type of fiber was expected to deliver a similar mechanical concrete performance.

## Figures and Tables

**Table 1:** FRC dosages used in the present study.

	Dosage FRCs (kg/m³)
Cement II 42.5R	325
Crushed limestone gravel (6–10 mm)	430
Crushed limestone gravel (4–6 mm)	580
Natural sand (0–4 mm)	835
Limestone Filler	80
Water	178.5
Superplasticizer	0.70% ± 0.2%

**Table 2:** Properties of the fibers used in the present study *.

Code	Type	Length l_f_ (mm)	Diameter d_f_ (mm)	Aspect Ratio	Melting Point (°C)	Tensile Strength (MPa)	Density (kg/m³)
P1	Macrosynthetic	35	1.00	35	170	550	920
P2	Macrosynthetic	54	0.50 ^1^	108	160	600	910
S	Steel	48	0.55	87	1375	1115	7850

^1^ This is the measure of a single fiber. The bundle diameter is 3.00 mm. * More specifications about these products can be found, respectively, at (https://esp.sika.com/es/products.html (last accessed: 23 June 2021)), (https://https:www.oscrete.com/uploads/datasheets/2020/03/Forta_Ferro.pdf (last accessed: 23 June 2021)) and (https://www.bekaert.com/es-MX/productos/construccion/refuerzo-de-hormigon/fibras-de-acero-dramix-3d-para-refuerzo-de-hormigon (last accessed: 23 June 2021)).

**Table 3:** The conservation conditions.

Code	Description	Characteristics of Conservation Condition	Image
05	Cool box Asfri Stock 100 (203 L)	5 °C	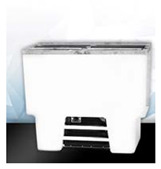
20	Humidity Chamber	20 °C, RH 95%	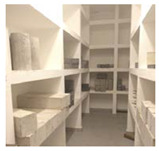
35	Thermal bath (480 L)	35 °C, specimens were submerged	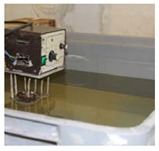
50	Thermal bath (580 L)	50 °C, specimens were submerged	

**Table 4:** Summary of the experimental program implemented.

Fiber Type	Pre-Cracking Age (Days)	Final Test Age (Days)	Batch	Temperature (°C)	Code	# of Specimens
P1	Not pre-cracked	60	B1	05	P1-N-05	4
				20	P1-N-20	4
			B2	35	P1-N-35	4
				50	P1-N-50	4
	28		B3	05	P1-P-05	4
				20	P1-P-20	4
			B4	35	P1-P-35	4
				50	P1-P-50	4
P2			B5	05	P2-P-05	4
				20	P2-P-20	4
			B6	50	P2-P-50	4
S			B7	05	S-P-05	4
			B8	20	S-P-20	4
				50	S-P-50	4

**Table 5:** Consistency class and compressive strength of all batches.

Batch	Code	Consistency Class	Compressive Strength	
			f_c,m_ (MPa)	CV (%)
B 1	P1-N-05 P1-N-20	S2	35.05	1.89
B 2	P1-N-35 P1-N-50	S2	37.26	2.49
B 3	P1-P-05 P1-P-20	S2	35.68	2.39
B 4	P1-P-35 P1-P-50	S2	35.42	1.28
B 5	P2-P-05 P2-P-20	S1	35.20	1.22
B 6	P2-P-50	S1	36.47	2.53
B 7	S-P-05	S3	31.60	5.80
B 8	S-P-20 S-P-50	S3	32.73	1.46

**Table 6:** Results of the residual flexural strengths obtained at moderate temperatures for uncracked specimens (60 days).

Code	P1-N			
	5 °C	20 °C	35 °C	50 °C
$f_{L}\;$ (MPa)	4.28	58	4.69	4.24
CV (%)	11.37	12.61	9.97	14.36
$f_{R,a}\;$ (MPa)	2.01	39	2.04	2.26
CV (%)	9.20	15.36	36.14	19.71
$f_{R,b}\;$ (MPa)	3.00	18	3.13	4.04
CV (%)	12.35	13.94	38.44	34.84

**Table 7:** Results of the residual flexural strengths obtained at moderate temperatures for pre-cracked specimens.

Code	Age of Testing	P1-P				P2-P			S-P		
		5 °C	20 °C	35 °C	50 °C	5 °C	20 °C	50 °C	5 °C	20 °C	50 °C
$f_{L}\;$ (MPa)	28 days	3.67	4.15	3.98	4.24	3.91	4.36	5.10	4.43	4.27	4.36
CV (%)		4.23	9.40	29.76	2.78	8.65	6.85	6.59	6.73	3.43	2.64
$f_{R,a}\;$ (MPa)		2.05	1.45	1.60	1.40	1.26	1.79	1.84	2.31	2.95	2.38
CV (%)		3.32	33.66	21.69	7.22	10.34	38.69	25.93	4.74	18.96	17.20
$f_{R,b}\;$ (MPa)	60 days	3.52	3.14	2.26	2.23	1.86	3.14	2.33	2.35	3.51	2.69
CV (%)		6.53	18.36	15.76	13.57	10.64	41.36	29.93	26.43	26.94	18.84

**Table 8:** Toughness indices of the analyzed concretes.

Code	I_5_	CV (%)	I_10_	CV (%)	I_20_	CV (%)
P1-N-05	4.38	11.57	7.80	13.46	15.70	15.37
P1-N-20	3.69	41.83	6.41	40.87	12.50	41.04
P1-N-35	4.47	25.50	7.99	37.80	16.96	52.44
P1-N-50	3.11	22.09	5.62	27.08	12.28	32.25
P1-P-05	4.10	12.01	9.71	15.50	19.15	16.52
P1-P-20	5.14	18.41	9.35	32.00	19.49	36.60
P1-P-35	4.67	9.25	9.87	5.37	14.94	64.62
P1-P-50	4.17	4.92	10.71	28.79	18.68	26.12
P2-P-05	3.73	10.93	5.99	11.50	10.94	12.69
P2-P-20	3.95	26.14	9.75	43.61	14.75	18.89
P2-P-50	3.90	12.74	7.41	22.78	11.65	29.30
S-P-05	4.33	11.96	7.70	11.93	14.90	4.83
S-P-20	4.79	5.65	9.68	8.64	20.02	12.91
S-P-50	3.68	12.31	7.06	14.66	13.78	17.36

**Table 9:** Effect of P1 fiber type and temperature on $f_{L}$ by applying ANOVA analysis.

Source	Square Sum (SS)	DF	Mean Square (MS)	F-Value	*p*-Value
Factor (Between)	1.49085	3	0.49695	7.68	0.0059
Error (Within)	0.647242	10	0.0647242		
Total	2.13809	13			

**Table 10:** Effect of P2 fiber type and temperature on $f_{L}$ by applying ANOVA analysis.

Source	Square Sum (SS)	DF	Mean Square (MS)	F-Value	*p*-Value
Factor (Between)	3.15172	2	1.57586	15.15	0.0013
Error (Within)	0.93615	9	0.104017		
Total	4.08787	11			

**Table 11:** Effect of S fiber type and temperature on $f_{L}$ by applying ANOVA analysis.

Source	Square Sum (SS)	DF	Mean Square (MS)	F-Value	*p*-Value
Factor (Between)	0.0582	2	0.0291	0.72	0.5122
Error (Within)	0.363025	9	0.0403361		
Total	0.421225	11			

**Table 12:** Effect of P1 fiber type and temperature on $f_{R,a}$ by applying ANOVA analysis.

Source	Square Sum (SS)	DF	Mean Square (MS)	F-Value	*p*-Value
Factor (Between)	0.840602	3	0.280201	2.13	0.1603
Error (Within)	1.31748	10	0.131748		
Total	2.15809	13			

**Table 13:** Effect of P2 fiber type and temperature on $f_{R,a}$ by applying ANOVA analysis.

Source	Square Sum (SS)	DF	Mean Square (MS)	F-Value	*p*-Value
Factor (Between)	0.879017	2	0.439508	2.12	0.1756
Error (Within)	1.86235	9	0.206928		
Total	2.74137	11			

**Table 14:** Effect of S fiber type and temperature on $f_{R,a}$ by applying ANOVA analysis.

Source	Square Sum (SS)	DF	Mean Square (MS)	F-Value	*p*-Value
Factor (Between)	1.06622	2	0.533108	2.41	0.1452
Error (Within)	1.99115	9	0.221239		
Total	3.05737	11			

**Table 15:** Effect of P1 fiber type and temperature on $f_{R,b}$ by applying ANOVA analysis.

Source	Square Sum (SS)	DF	Mean Square (MS)	F-Value	*p*-Value
Factor (Between)	4.94657	3	1.64886	11.86	0.0009
Error (Within)	1.52917	11	0.139015		
Total	6.47573	14			

**Table 16:** Effect of P2 fiber type and temperature on $f_{R,b}$ by applying ANOVA analysis.

Source	Square Sum (SS)	DF	Mean Square (MS)	F-Value	*p*-Value
Factor (Between)	3.53105	2	1.766552	2.37	0.1485
Error (Within)	6.69125	9	0.743472		
Total	10.2223	11			

**Table 17:** Effect of S fiber type and temperature on $f_{R,b}$ by applying ANOVA analysis.

Source	Square Sum (SS)	DF	Mean Square (MS)	F-Value	*p*-Value
Factor (Between)	2.94935	2	1.47468	4.26	0.0499
Error (Within)	3.11565	9	0.346183		
Total	6.065	11			

## Data Availability

Not applicable.
